# Evaluation of estrogenic and anti-estrogenic activity of endocrine disruptors using breast cancer spheroids: a comparative study of T47D and MCF7 cell lines in 2D and 3D models

**DOI:** 10.3389/ftox.2025.1547640

**Published:** 2025-02-21

**Authors:** Katia Barbaro, Elisa Innocenzi, Valentina Monteleone, Daniele Marcoccia, Annalisa Altigeri, Alessia Zepparoni, Daniela Caciolo, Cristian Alimonti, Marta Mollari, Paola Ghisellini, Cristina Rando, Roberto Eggenhöffner, Maria Teresa Scicluna

**Affiliations:** ^1^ Istituto Zooprofilattico Sperimentale del Lazio e della Toscana “M. Aleandri”, Rome, Italy; ^2^ Department of Surgical Sciences and Integrated Diagnostics (DISC), Genova University, Genova, Italy

**Keywords:** endocrine disruptors, estrogen receptor signaling, bisphenol A, 2D/3D *in vitro* models, breast cancer toxicological assessment

## Abstract

**Introduction:**

The estrogenic and anti-estrogenic effects of endocrine disruptors were examined *in vitro* using two-dimensional 2D and three-dimensional 3D estrogen receptor-positive T47D and MCF7 human breast cancer cells.

**Methods:**

The *in vitro* model system was used to test the plasticizer Bisphenol A (BPA), a known endocrine disruptor (EDs) with estrogen-like action, aga inst 17β-Estradiol (E2), the endogenous nuclear estrogen receptor (nERs) ligand, and the anti-estrogenic drug Fulvestrant (FUL). Spheroid formation and gene expression of estrogen-regulated markers (pS2 and TGFβ3) both in 2D and 3D cultures were used to establish the dose-dependent cellular effects of these substances, evaluated cell viability either by separately treating with the individual substances or in co-treatment.

**Results:**

BPA exhibited a dose-dependent estrogenic activity in both 2D and 3D cultures, significantly influencing cell proliferation and gene expression of estrogen-regulated markers (pS2 and TGFβ3). In contrast, FUL displayed anti-estrogenic properties, effectively inhibiting the proliferative effects of E2, thereby highlighting the complex interactions between these compounds and the nERs pathways in human breast cancer cells.

**Discussion:**

Our findings indicate that E2 and BPA significantly increase pS2 expression while decreasing TGFβ3, and that FUL co-treatment reverses these effects. Therefore, the *in vitro* model system could serve to observe the cell-mediated effects caused by the interaction of EDs with nERs. Through the use of these *in vitro* model systems - 2D and especially 3D, the latter of which allow better emulation of complex physiological and pathological processes occurring *in vivo*, the effects caused by EDs on nERs pathways can be detected and studied under various conditions. This approach performs as a preliminary screening tool to identify estrogenic substances, offering the potential to reduce reliance on *in vivo* experiments and contributing to improved environmental and health risk assessments.

## 1 Introduction

A severe public health concern is represented by the so-called endocrine disruptors (EDs) interfering with hormonal processes and the endocrine system in humans (Solecki et al., 2017; Corti et al., 2022; Tassinari et al., 2023; Galli et al., 2024). Among these substances, the estrogenic activity of Bisphenol A (BPA) is of particular concern (Vandenberg, 2019). The potential role of BPA in hormone-related diseases, such as reproductive issues and hormone-dependent cancers, raises serious alarms due to its ability to mimic estrogen activity (Rochester, 2013), the environmental impact of EDs can be significant in view of their widespread presence, persistence, and potential effects on living organisms (Rubin, 2011; Kumar et al., 2020). One of the priorities of the European Commission (EC) is, therefore, the development of new *in vitro* analytical screening methodologies for efficient environmental monitoring and a deeper understanding of action and toxicity mechanisms of EDs (Grignard et al., 2021; Braeuning et al., 2023). The present contribution, in the absence of a formal optimized methodology for efficient bioactivity study of EDs, describes an ultrasensitive analytical methodology developed to deliver, with high precision and reliability, information on the behavior of these substances and their interaction with cellular systems. For estrogenic activity, BPA was analyzed in comparison with 17β-estradiol (E2), being the endogenous ligand acting on estrogen receptors, and Fulvestrant (FUL), a known anti-estrogenic drug (Heldring et al., 2007; Guan et al., 2019). Nuclear receptor activation by E2 forms a basis for estrogenic activity, and the antagonistic actions of FUL illustrate how nERs signaling can be selectively inhibited. Together, these substances allow comparison of the actions of BPA with endogenous and therapeutic modulators of estrogenic pathways (Seachrist et al., 2016; Chakraborty et al., 2023). The methodology is in alignment with the ethical and scientific imperative to reduce animal testing, as required by the European Directive 2010/63/EU (EU, 2010), by promoting the use of alternative methods in line with the principles of replacement, reduction, and refinement (3R) (Lorenzetti et al., 2015). *In vitro* models using human cell lines could be used to determine the adverse effects caused on human health by exposure to EDs (Yilmaz et al., 2020), also allowing the study of molecular mechanisms that cannot be addressed in animal models (Taylor, 2019). Our study moves on these recent observations by using human breast cancer-derived cell lines, T47D and MCF7, which express high levels of ERα and ERβ, making them sensitive models for the evaluation of estrogenic and anti-estrogenic compounds (Martinez-Bernabe et al., 2021). The purpose of this work is to evaluate cellular responses to BPA, E2, and FUL by determining the gene expression of estrogen-regulated markers (pS2 and TGFβ3) in an *in vitro* in both two-dimensional (2D) and three-dimensional (3D) breast cancer cell model. The first of these estrogen-regulated markers, pS2, is a well-established marker of ER activation (Krieg et al., 2004); the second, TGFβ3, allows evaluating cellular differentiation and tumor suppressive mechanisms (Liu et al., 2021). Thus, estrogenic and anti-estrogenic activity can be evaluated in controlled experimental conditions, allowing for precise assessment of how BPA influences nER pathways, either by mimicking natural estrogen or by inhibiting its effects, thereby affecting estrogen-regulated biological processes (Gao et al., 2015; Kim et al., 2015). In addition, by using 2D and 3D cultures (Edmondson et al., 2014), our aim is to compare *in vitro* the 2 cell systems, in particular using the 3D model the structural and cell-cell interaction conditions represent more closely the human tissue, which cannot be replicated with a simple 2D monolayer culture. Finally, this study aims to propose an *in vitro* approach that could be used as a preliminary screening system for the determination of EDs with estrogenic and/or anti-estrogenic-like activity through the study of gene markers respectively in 2D and 3D cell cultures.

## 2 Materials and methods

### 2.1 Chemicals

The chemicals used in this study include E2, the endogenous estrogen and ER-agonist (CAS no. 50-28-2, purity ≥98% Sigma-Aldrich, Munich, Germany); BPA a plasticizer and ER-agonist (CAS no. 80-05-7, purity ≥99% Sigma-Aldrich, Munich, Germany) and FUL a pharmacological drug and ER-antagonist (CAS no. 129453-61-8, purity >98%, Sigma-Aldrich, Munich, Germany). All solvents were high-performance liquid chromatography (HPLC) grade, and water was ultrapure grade.

### 2.2 Cell culture 2D and reagents

Cell culture reagents DMEM with/without (w/o) phenol red, fetal bovine serum (FBS) and charcoal-stripped (CS-FBS) were purchased from Gibco (Invitrogen Life Technologies, San Giuliano Milanese, Italy) except for penicillin and streptomycin (PS) and dimethyl sulfoxide (DMSO) both bought from Sigma-Aldrich. T47D cells (derived from the pleural effusion of a ductal human carcinoma of the breast) were purchased from ATCC (American Type Culture Collection/LGC Standards SRL). The MCF7 cells were derived from the pleural effusion of a Caucasian female, suffering from a breast adenocarcinoma and kindly provided by the former laboratory of Professor Loreni Fabrizio of the University of Tor Vergata, Rome Italy. MCF7 cells were grown in; DMEM phenol red complete medium (growth medium/GM with FBS 10%) & Treatment Medium containing DMEM without phenol red and 5% CS- FBS); all chemicals were dissolved in DMSO to obtain 100 mM stock solutions. Working stock solutions and dilutions were prepared in 1x GM without FBS and PS just before use to always dilute vehicle (DMSO) concentration to 0.01%. Vehicle treated cells (0.01% DMSO) were used as controls (CTRL) in all experiments, which were conducted as three biological replicates. All cells were maintained in a humidified incubator at 37°C with 5% CO_2_ concentration. The cytotoxic impact, both alone and in co-treatment of the substances selected for the study, was evaluated on T47D and MCF7 cell cultures in monolayer by MTT (3-[4,5-dimethylthiazol-2-yl]-2,5 diphenyl tetrazolium bromide) assay. Before treatments, the T47D and MCF7 cell lines were plated at a density of 10 × 10^3^ cells/well in 96-well plates in 200 μL TM (Treatment Medium containing DMEM without phenol red and 5% CS- FBS) and incubated for 24 h. Subsequently, the cell lines were then treated for 24 h with the study substances to perform a dose-response curve from 1 pM to 100 μM before assessing cytotoxicity. Likewise, for gene expression, before treatments, T47D and MCF7 cells were plated in 12.5 cm^2^ flasks (8 × 10^5^ cells/flask) and incubated for 24 h in TM, then washed with 1× phosphate buffer solution/PBS, pH 7.2 w/o Ca^2+^ and Mg^2+^ and treated for 24 h with the steroid hormone E2, the plasticizer BPA in the presence or absence of FUL in TM.

### 2.3 Gene expression analysis by RT-PCR

Gene expression analysis was performed using PureLink RNA Mini kit (Invitrogen, USA), cDNA High-Capacity cDNA Reverse Transcription kit and Powerup SYBR Green PCR Master Mix by Applied Biosystems; employing oligonucleotide primer sequences designed by Kim and Park (2013) and purchased from Carlo Erba Reagents (Italy). The selected primer sequences were reported in the following Table 1.

**TABLE 1:** Sequences of primers for the RT-PCR.

Gene symbol (mRNA)	Primer	Sequence (5′→ 3′)
*GAPDH*	Forward	TGG​GCT​ACA​CTG​AGC​ACC​AG
	Reverse	GGG​TGT​CGC​TGT​TGA​AGT​CA
*pS2*	Forward	CCA​CCA​TGG​AGA​ACA​AGG​TGA
	Reverse	GCA​GCC​CTT​ATT​TGC​ACA​CTG
*TGFβ3*	Forward	ATG​AGC​ACA​TTG​CCA​AAC​AGC
	Reverse	CAC​TCA​CGC​ACA​GTG​TCA​GTG​A

### 2.4 Cell culture 3D and reagents

T47D and MCF7 cell lines were also employed to generate tumor spheroids 3D. Materials used to maintain the 3D cultures include DMEM-F12 w/o phenol red, CS-FBS acquired from Gibco (Invitrogen Life Technologies, San Giuliano Milanese, Italy) and PS and DMSO both purchased by Sigma-Aldrich. T47D and MCF7 cells were expanded using 2D cell cultures and were grown for 1 1 week in DMEM-F12-based complete medium with 10 % CS-FBS and 1% PS. Subsequently, the aforementioned cell lines were maintained in starvation for 24 h in DMEM-F12-based complete medium with 5% CS-FBS and 1% PS. After this time, 4 × 10^3^ cells per well were plated in a 96-well U-bottom (650970-Greiner bio-one), clear, cell-repellent surface, lid with condensation rings, microplate in the same type of complete medium as previously mentioned and centrifuged at 250 g for 5 s. After 48 h of growth, the 3D cultures were treated for 24 h with both the addition of the estrogenic substances (E2 and BPA) and the anti-estrogenic drug FUL subsequently extracted mRNA utilizing the MagPurix Viral Kit (Zinexts Life Science Corp, United Kingdom). Gene expression analysis was performed using the kits and oligoprimers previously given above.

### 2.5 Statistical analysis

All data were obtained from three independent experiments performed in triplicate. Cytotoxicity data was expressed as mean ± S.D. and analyzed by the non-parametric Dunnett test for multiple comparisons (software Sas Jmp Statistical Discovery v14.0, Milan, Italy). Gene expression, expressed as arbitrary units upon normalization with the reference housekeeping gene GAPDH, data were expressed as mean ± S. D and analyzed by Student’s t-test (software Sas Jmp Statistical Discovery v14.0, Milan, Italy).

Throughout the study, P-values ≤0,05; ≤0,01; and ≤0.001 were considered statistically significant, as indicated in Figure legends.

### 2.6 Image data acquisition

Growth of T47D and MCF7 spheroids was measured every day for 5 days using a Nikon Eclipse TE2000-U light microscope with a built-in camera, Nikon Digital Sight 10. Images were captured at ×10 magnification, and measurements were taken using the NIS Elements D 5.41.02 program, ensuring that all measurements are done using the “Annotations and Measurements” option to have accurate data from day one to day five.

## 3 Results and discussions

### 3.1 Cell 2D viability and indirect cell proliferation

**FIGURE 1:**
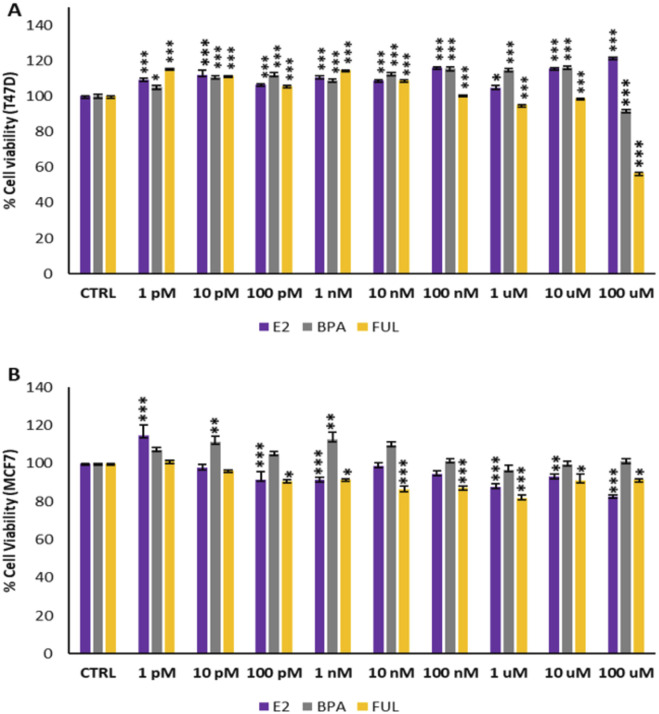
**(A, B)** dose–response curves of T47D and MCF7 cells viability after treatments with the estrogenic substances (E2 and BPA) and anti-estrogenic drug (FUL). Data are expressed as percentage values (±S.D.) in comparison to CTRL values corresponding to 100% and represent the mean values of three independent experiments performed in triplicate. P-values (Dunnett test): p < 0.05 *, p < 0.01 **, p < 0.001 *** vs. CTRL.

Figures 1A, B depicts the dose–response curves for T47D and MCF7 cell viability after treatments with E2, BPA, and FUL. As illustrated in Figure 1, Panel A, there was a statistically significant and dose-dependent increase in cell viability observed for the T47D cell line across all treatment concentrations of both E2 and BPA. The highest estrogenic effect is given by both E2 and BPA, in particular at concentrations above 100 nM, showing that these estrogenic compounds stimulate T47D cells, a direct confirmation of their responsivity as estrogen receptors. However, for FUL, the increase in cell viability was statistically significant only up to a concentration of 10 nM, with a notable decrease in cell viability at higher concentrations (ranging from 100 nM to 100 μM). These results show that FUL acts effectively as an anti-estrogenic agent although in a concentration-dependent way. In Figure 1, Panel B, no drastic cytotoxic effects were noted in the MCF7 cell line for E2, BPA, or FUL. However, a statistically significant slight decrease in cell viability was observed starting at a concentration of 100 pM for both E2 and FUL. In contrast, BPA treatment resulted in an increase in cell viability at all concentrations, particularly at 10 pM and 1 nM, which were statistically significant.

### 3.2 pS2 and TGFβ3 expression analysis in 2D cell lines

**FIGURE 2:**
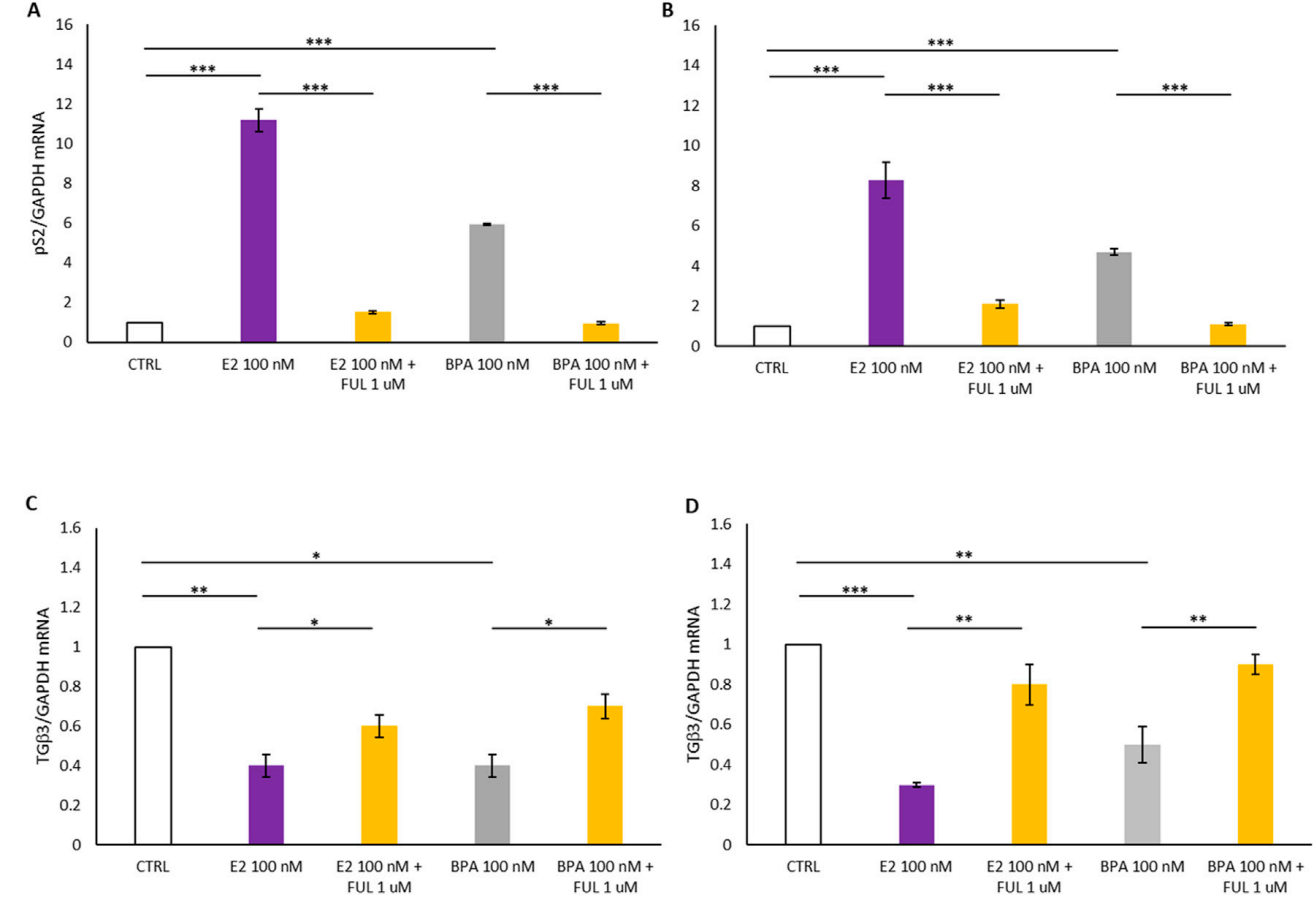
**(A–C)** shows the effects of 17β-Estradiol and Bisphenol A with and without co-treatment with Fulvestrant for 24 h on the expression of *pS2* and *TGFβ3* mRNA in T47D cells. **(B–D)** shows the effects of 17β-Estradiol and Bisphenol A with and without co-treatment with Fulvestrant for 24 h on the expression of *pS2* and *TGFβ3* mRNA in MCF7 cells. Data are expressed as 2^−ΔΔCT^ (±S.D.) and represent mean values of three independent experiments performed in triplicate. P-values (t-test): p < 0.05 *, p < 0.01 **, p < 0.001 *** vs. CTRL; p < 0.05 *, p < 0.01 **, p < 0.001 *** vs. E2 100 nM and p < 0.05 *, p < 0.01 **, p < 0.001 *** vs. BPA 100 nM.

Figure 2 illustrates the mRNA expression levels of pS2 and TGFβ3 in T47D and MCF7 cell lines treated with E2 and BPA, with and without co-treatment with FUL, over a 24-h period. Panels A and B of Figure 2 are indicative of a statistically significant increase in pS2 levels following treatment with 100 nM E2 and BPA 100 nM in T47D (Panel A) and MCF7 (Panel B) breast cancer cells compared to the CTRL. In contrast, co-treatment with FUL significantly inhibited the estrogenic effects of E2 and BPA when compared to treatments with E2 or BPA alone. As shown in Panels C and D in Figure 2, the treatment with 100 nM E2 and BPA 100 nM induced a statistically significant decrease in TGFβ3 expression in the treated cells compared to CTRL (Figure 2, Panels C–D), suggesting that these treatments suppress the expression of this differentiation marker. Conversely, co-treatment with FUL resulted in a significant increase in TGFβ3 expression compared to cells treated only with E2 and BPA.

### 3.3 Formation of T47D and MCF7 spheroids

**FIGURE 3:**
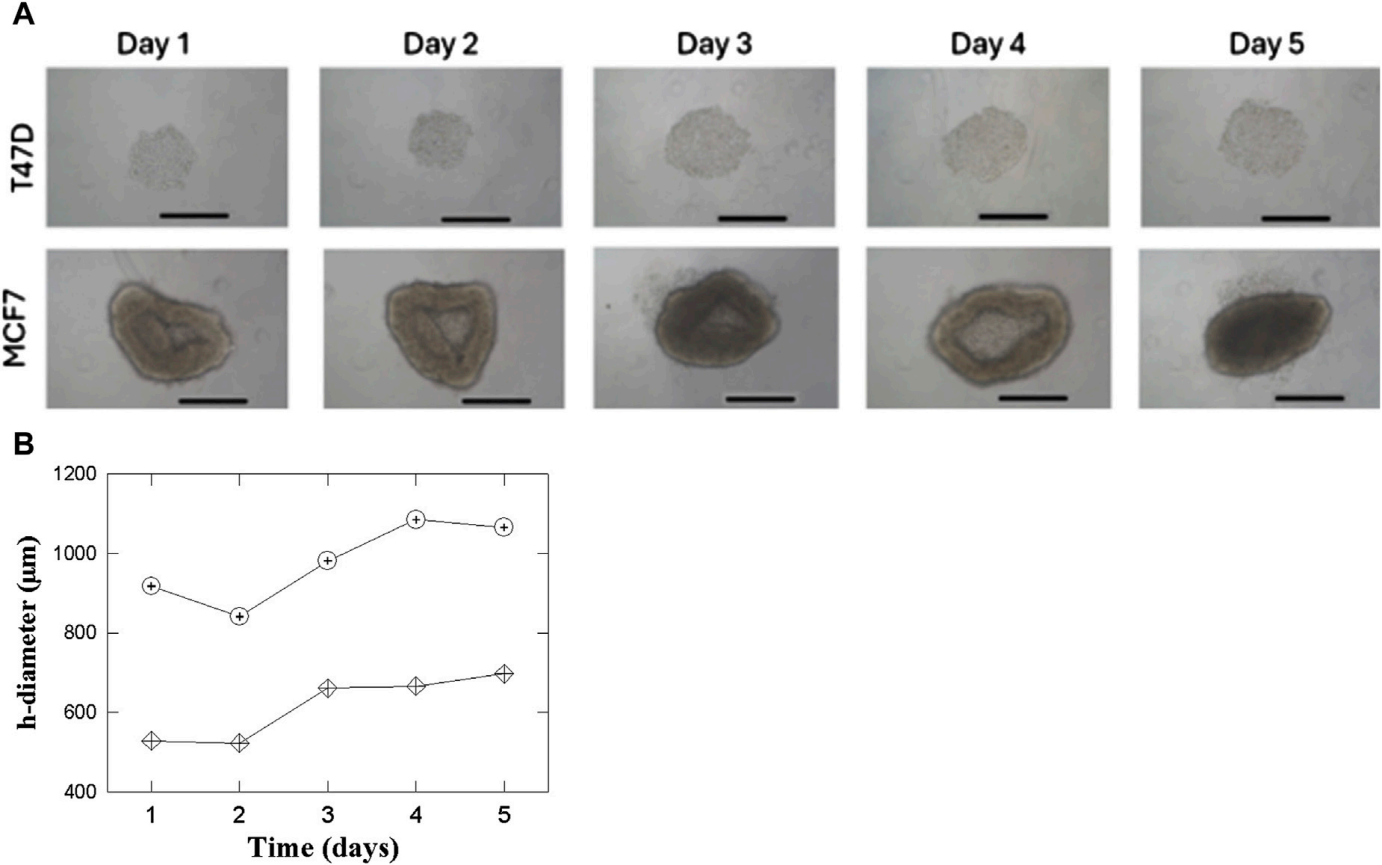
**(A)** T47D and MCF7 cell spheroids growth in a 96 well round bottom. 4 × 10^3^ cells/well were seeded. ×10 microscope magnification was used between days 1 and 5. The length of the bars in all the Panels corresponds to 500 µm. **(B)** Behavior of the horizontal diameter for the 2 cell lines T47D (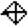) and MCF7 (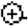) between days 1 and 5.

The cell lines T47D and MCF7 successfully formed spheroids, spontaneously. However, their shape is different between T47D and MCF7. The former (images at the top of the figure) give rise to mostly 3D rounded shapes that appear compact and dense growing from day 1 to day 5 (Figures 3A, B). The uniform and dense shape can indicate strong inter-cell adhesion. The behavior shown by MCF7 (bottom images) is quite different. They also aggregate to form spheroids, but their shape is larger and more irregular than in T47D spheroids.

Figure 3B quantifies the increase in the horizontal diameter of T47D and MCF7 spheroids over 5 days to supplements the images in Figure 3A. The graph shows that though both cell lines exhibit net growth, the horizontal diameter of both types of spheroids decreases on the second day. This may be caused by initial readjustments or even contraction of the cells within the spheroids as the culture equilibrates after formation. After this initial reduction, the T47D spheroids steadily and progressively increase in size, mirroring their reproducible and dense growth. The MCF7 spheroids have a greater increase in x-axis diameter after Day 2, in line with their larger and less dense growth appearance as illustrated by Figure 3A.

### 3.4 pS2 and TGFβ3 expression analysis in 3D cell lines

**FIGURE 4:**
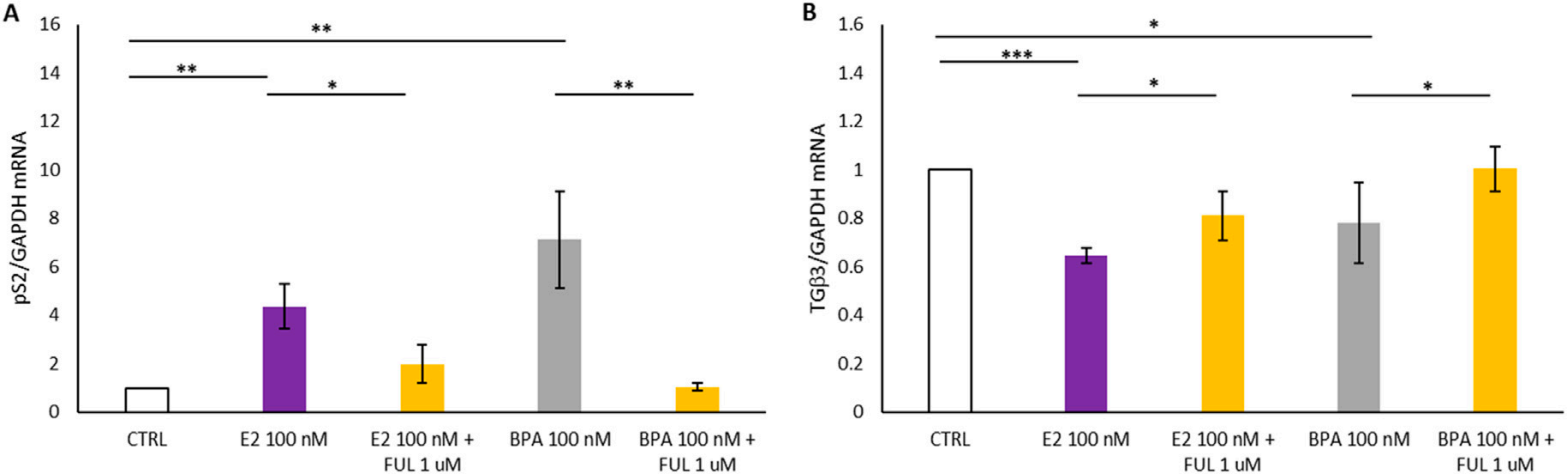
**(A, B)** Shows the effects of 17β-Estradiol and Bisphenol A with and without co-treatment with Fulvestrant for 24 h on the expression of *pS2* and *TGFβ3* mRNA in T47D cells spheroides. Data are expressed as 2^−ΔΔCT^ (±S.D.) and represent mean values of three independent experiments performed in triplicate. P-values (t-test): p < 0.05 *, p < 0.01 **, p < 0.001 *** vs. CTRL; p < 0.05 *, p < 0.01 **, p < 0.001 *** vs. E2 100 nM and p < 0.05 *, p < 0.01 **, p < 0.001 *** vs. BPA 100 nM.

In Figure 4, Panel A shows that the treatment with E2 at 100 nM and BPA 100 nM resulted in a statistically significant increase in the expression of the estrogen-responsive pS2 gene in T47D spheroids compared to the control (CTRL): it turns out upregulated by the activation of the nERs. In contrast, co-treatment with FUL at 1 µM significantly inhibited the estrogenic effects of E2 and BPA when compared to treatment with E2 or BPA alone: when the nERs -antagonist FUL is added, pS2 genes are inactivated and its expression drops. Additionally, the behavior of the TGFβ3 gene associated with cell differentiation is studied in the 3D spheroid cultures of T47D breast cancer cells, and shown in Figure 4, Panel B. The treatment with E2 and BPA (100 nM) induced a statistically significant decrease in TGFβ3 expression in the treated spheroids compared with the CTRL. On the contrary, co-treatment with FUL at 1 µM significantly increased TGFβ3 expression compared to the spheroids of T47D treated only with E2 and BPA, restoring TGFβ3 expression closer to CTRL. This restoration proves that FUL acts not only by blocking the activation of the estrogen-responsive genes, such as pS2, but also counteracts the inhibition of the expression of TGFβ3 by E2 and BPA.

**FIGURE 5:**
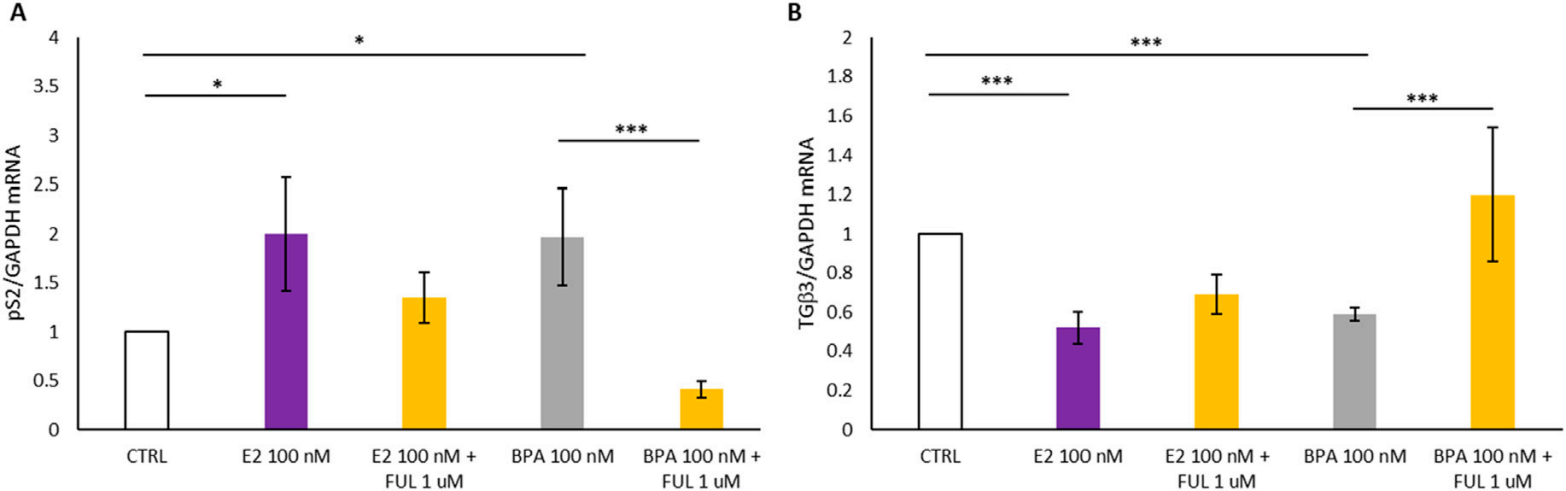
**(A, B)** Shows the effects of 17β-Estradiol and Bisphenol A with and without co-treatment with Fulvestrant for 24 h on the expression of *pS2* and *TGFβ3* mRNA in MCF7 cells spheroides. Data are expressed as 2^−ΔΔCT^ (±S.D.) and represent mean values of three independent experiments performed in triplicate. P-values (t-test): p < 0.05 *, p < 0.01 **, p < 0.001 *** vs. CTRL; p < 0.05 *, p < 0.01 **, p < 0.001 *** vs. E2 100 nM and p < 0.05 *, p < 0.01 **, p < 0.001 *** vs. BPA 100 nM.

Figure 5 reports how treatments with E2 and BPA, both with and without the addition of FUL, affect the expression of two genes, pS2 and TGFβ3, in 3D spheroid cultures of MCF7 breast cancer cells over a 24-h period. In Figure 5, Panel A, we observed a statistically significant increase in pS2 levels in MCF7 spheroids following treatment with 100 nM E2 and BPA 100 nM compared to the CTRL. This increase indicates that BPA can mimic the effects of E2 on the pS2 gene in MCF7 spheroids. Additionally, co-treatment with FUL at 1 µM significantly inhibited the estrogenic effects of BPA when compared to treatment with BPA alone. These results confirm that FUL, an estrogen receptor antagonist, effectively blocks the estrogenic effects of BPA on pS2 expression. By binding to nERs, FUL prevents BPA from activating the receptor, thereby reducing pS2 expression closer to control levels. In the same MCF7 spheroids, treatment with 100 nM E2 and BPA 100 nM led to a statistically significant decrease in TGFβ3 expression compared to CTRL, although in BPA less pronounced than in E2. Conversely, co-treatment with FUL resulted in a substantial increase in TGFβ3 expression compared to spheroids treated only with BPA, as shown in Figure 5, Panel B, indicating again its role in counteracting action.

Comparison can be carried out between Figure 4 for T47D spheroids and Figure 5 for MCF7 spheroids to expose the response of these two different cell lines to the E2, BPA, and FUL treatments in 3D culture. Considering pS2 expression (Panels A in both Figures), both the E2 and BPA (100 nM) significantly increased pS2 expression in both the T47D and MCF7 spheroids when compared to the CTRL, thus showing that both cell lines respond to such exposures through the activation of the estrogen-responsive gene pS2, both T47D and MCF7 cells recognize E2 and BPA as estrogenic agents, stimulating nER pathways in a similar manner. Conversely, FUL is effective in inhibiting BPA and E2-induced pS2 expression in both cell lines, but the inhibition appears stronger in T47D spheroids, suggesting T47D may be more responsive to FUL’s anti-estrogenic effects.

Relative to the expression of TGFβ3 (Panels B in both Figures 4, 5), E2 and BPA significantly decrease TGFb3 expression comparted to the CTRL in both MCF7 spheroids (Figure 5, Panel B) and T47D spheroids (Figure 4, Panel B). However, the suppression of TGFb3 is more pronounced in MCF7 compared to T47D spheroids, which show a less marked decrease. Notably, BPA elicited a weaker suppressive effect on TGFβ3 expression in T47D than in MCF7 spheroids, whereas E2 consistently reduced TGFβ3 expression to a similar extent in both cell lines. FUL’s ability to restore TGFβ3 expression is evident in both cell lines, but the effect is more pronounced in T47D spheroids. This might suggest that FUL’s differentiation-promoting effect through TGFβ3 restoration is stronger in T47D than in MCF7 cells. Both T47D and MCF7 spheroids respond to E2 and BPA with increased pS2 expression, indicating activation of estrogen receptor pathways. However, T47D cells appear slightly more sensitive to FUL’s inhibitory effects on pS2 than MCF7 cells. E2 and BPA decrease TGFβ3 expression in both cell lines, but this effect is stronger in T47D spheroids. This might suggest that T47D cells are more affected by estrogenic compounds in terms of differentiation and tumor-suppressive signaling (as reflected by TGFβ3). FUL effectively blocks the estrogenic effects of E2 and BPA in both cell lines, restoring baseline or near-baseline levels of pS2 and TGFβ3. However, the inhibitory effects of FUL are more pronounced in T47D, making this cell line potentially more useful for evaluating the anti-estrogenic effects of compounds. These results address some practical conclusions concerning the used of the 2D or 3D *in vitro* models system: T47D cells may be more suitable for studies focused on estrogen receptor-mediated effects due to their consistent and strong response to both estrogenic and anti-estrogenic treatments. MCF7 cells, while also responsive, show slightly less pronounced changes, indicating potential differences in receptor expression or pathway activation between the 2 cell lines. This comparison underlines the fact that while both T47D and MCF7 respond to the estrogenic and anti-estrogenic compounds, T47D might provide more expressive and robust data concerning estrogen receptor modulation in 3D culture models. As already presented above in Figures 3A,B, besides higher sensitivity towards the three selected compounds, the T47D cells form stable, compact spheroids, hence ideal for 3D *in vitro* model system. Their solid, consistent structure holds over time, allowing size and density measurements to be highly accurate and reliable. In view of the above-mentioned considerations, the T47D spheroid would have less shape variability and thus they would be more stable and uniform - ideal conditions for reliable drug testing In T47D spheroids, any statistically significant change in size or density would thus be attributed to the treatment under investigation, rather than to intrinsic structural changes within the spheroids themselves, which were observed in the MCF7 spheroids. The cell viability and gene expression measurements both serve valuable purposes, and each provides complementary information. In contrast to gene expression, cell viability assays represent a stable, more aggregate measure of the overall health of the cell population and, therefore, are generally less variable and highly reproducible. This stability is advantageous in those applications where consistency is central, such as drug screening or toxicity testing, where clear comparisons across treatments are often required. In addition, it minimizes the robustness of the data obtained in cell viability to variability, besides making sure that any observed change is owing to treatment effects *per se*, and not due to fluctuations caused by other conditions. It is, therefore, particularly useful for applications with a general cytotoxicity focus or survival. In turn, gene expression analysis gives a much more sensitive view of cellular responses at the molecular level. This will perhaps provide some insight into how treatments are interacting with the cellular pathways, particularly hormone receptor pathways, by studying specific genes such as pS2 and TGFβ3, and subtle effects may be identified which could not be accessed only by viability. Despite higher variability, given both the inherent complexity of transcriptional regulation and the sensitivity of Q-PCR, gene expression analysis did provide specific mechanistic insights into early molecular responses to compounds. Such sensitivity is indeed necessary in various applications, where either induction or repression of pathways is of prime interest, as in EDs studies or mechanistic drug response analyses.

## 4 Conclusion

This study has demonstrated that T47D and MCF7 cell lines of breast cancer both represent a valid *in vitro* 3D model for the evaluation of estrogenic and anti-estrogenic activities of EDs, such as BPA, as they are able to represent the complex physiological and pathological processes occurring *in vivo*, due to the effects caused by EDs on nERs alteration pathways that can be detected. The results mainly underline the value of T47D cells, whose spheroids are more stable and compact, showing minor structural variation and hence being better as 3D *in vitro* model system. The T47D spheroids were uniform in composition, allowing precise and reproducible measurements for size, and gene expression; thus, they offer a better model for high-sensitivity testing. In particular, T47D and MCF7 spheroids were comparably highly inducing pS2 genes with low expression of TGFβ3 in 3D cultures in response to E2 and BPA treatments, indicating an estrogenic effect. However, T47D spheroids were more sensitive to FUL, evidenced by the higher levels of the inhibition of the induction of pS2 and the restoration of TGFβ3 levels, reflecting increased sensitivity to anti-estrogenic action. These results can be compared with the 2D data in Section 3.2: in the 2D model, both cell lines demonstrated high induction of pS2 and repression of TGFβ3 on their exposures to E2 and BPA. The manifestations were overridden by FUL. The expressions were less impressive in 2D than in the 3D model, especially regarding the expression of TGFβ3, and underlined the inability of 2D cultures to reproduce completely in 3D systems regarding the complexity of cellular interactions and microenvironments. This comparison with 2D results brings into perspective some benefits of 3D spheroid models, in which T47D cells are compactly growing and are healthy in their molecular response to treatments. These conditions make the 3D system more physiologically relevant for studying estrogenic and anti-estrogenic activities of endocrine disruptors such as BPA or therapeutic drugs such as FUL.

Two gene markers identified in this study were found as being useful in detecting the estrogenic/anti-estrogenic activities of compounds in both cell lines are pS2 and TGFβ3. BPA and E2 strongly induce the expression of pS2, demonstrating their estrogenic action, and concomitantly inhibiting the expression of TGFβ3, a gene associated with cellular differentiation and tumor suppression. The anti-estrogenic compound FUL strongly represses these activities, thereby restoring TGFβ3 and inhibiting pS2. This modulation thus underlines both the sensitivity and specificity of this assay in distinguishing between estrogenic stimulation and anti-estrogenic inhibition. In addition, the stronger response of T47D cells to BPA and FUL reinforces their suitability for detailed studies on estrogen receptor pathways and places them as a robust model in environmental testing and pharmacology. These findings will profoundly impact environmental and health risk assessments. The sensitivity and reproducibility of the T47D spheroid model meet present regulatory and ethical requirements for a reduction in animal testing by providing reliable *in vitro* testing alternatives. The cell model would thus be an effective tool in understanding biological effects and potential health risks associated with exposure to EDs chemicals and could be potentially used as the foundation for a high-throughput database on environmental chemicals in non-animal testing policy frameworks. In addition, this research contributes to the broad field of drug testing and toxicology by establishing T47D spheroids as a stable and consistent model in the screening of estrogenic and anti-estrogenic compounds. The capability of this *in vitro* model to provide reproducible data on endocrine activity justifies its use in toxicology and pharmacology, providing new perspectives toward better toxicological testing in line with the 3R principles of replacement, reduction, and refinement. The T47D 3D spheroid model in the end will provide a reliable, reproducible, and ethically compliant technique to evaluate EDs, with considerable potential for impact on regulatory science, furthering our knowledge in health effects related to environmental estrogens.

## Data Availability

All the relevant data are reported in the paper, requests for additional materials can be obtained from the corresponding author.
